# 基于知识导向的拟靶向分析方法建立及在糖尿病视网膜病变中的应用

**DOI:** 10.3724/SP.J.1123.2025.02011

**Published:** 2026-03-08

**Authors:** Yanqing HOU, Xiangyu CHI, Tinghu DU, Zengqi YAN, Daidi HOU, Chunxiu HU, Yuexing LIU, Xinyu LIU, Guowang XU

**Affiliations:** 1.大连理工大学化学学院，辽宁 大连 116024; 1. School of Chemistry，Dalian University of Technology，Dalian 116024，China; 2.中国科学院大连化学物理研究所，辽宁省代谢组学重点 实验室，辽宁 大连 116023; 2. Liaoning Province Key laboratory of Metabolomics，Dalian Institute of Chemical Physics，Chinese Academy of Science，Dalian 116023，China; 3.上海交通大学医学院附属第六人民医院糖尿病研究所，上海 200233; 3. Shanghai Sixth People’s Hospital Affiliated to Shanghai Jiao Tong University School of Medicine，Shanghai Diabetes Institute，Shanghai 200233，China; 4.天津威高医疗科技有限公司，天津 300500; 4. Tianjin Weigao Medical Technology Co. ，Ltd. ，Tianjin 300500，China; 5.中国医科大学，辽宁 沈阳 110122; 5. China Medical University，Shenyang 110122，China

**Keywords:** 糖尿病视网膜病变, 代谢组学, 超高效液相色谱-串联质谱, 疾病标志物, diabetic retinopathy （DR）, metabolomics, ultra-high performance liquid chromatography-tandem mass spectrometry （UHPLC-MS/MS）, disease markers

## Abstract

糖尿病视网膜病变（diabetic retinopathy，DR）是由糖尿病引起的常见致盲性眼病，也是全球成年人获得性视力丧失的主要原因。DR早期无症状，患者常因视力受损后再就诊而失去最佳治疗时机。传统DR检查方法存在局限性，不利于大规模快速筛查。生物标志物具有特异性和敏感性，能反映疾病所处阶段，寻找潜在生物标志物对DR早期诊断意义重大。本研究基于知识导向策略，从文献中筛选出142个与DR相关的潜在生物标志物，根据其物理化学性质，采用Merck Supelco Discovery HS F5色谱柱（100 mm×2.1 mm， 3 μm）分离代谢物，建立了基于超高效液相色谱-串联质谱（UHPLC-MS/MS）的正、负离子切换扫描分析的拟靶向分析方法，提高了分析的覆盖度和通量。以8种同位素内标对方法学进行考察，结果显示其在正、负离子模式下线性关系良好，线性范围达3个数量级以上，相关系数（*r* ^2^）均在0.995以上；3个浓度水平下提取回收率为75%~108%，相对标准偏差（RSD）小于13%；在正、负离子模式下，3个浓度水平下所有同位素内标的日内精密度RSD小于5%的个数占总数的91%，日间精密度RSD小于10%的个数占总数的91%，RSD最高不超过16.3%，表明方法精密度良好。对137例血清样本进行分析，筛选出85个差异代谢物，进一步分析发现胆碱和12-羟基二十碳四烯酸作为组合标志物能有效区分DR和无糖尿病视网膜病变的糖尿病患者。本研究开发的基于知识导向策略的拟靶向代谢组学分析方法为DR筛查和诊断提供了参考依据。

## Abstract (en)

Diabetic retinopathy （DR） is a common blinding eye disease caused by diabetes mellitus and is the leading cause of acquired vision loss in adults worldwide. DR is asymptomatic in its early stages， and patients often miss the optimal treatment window by the time they seek medical attention due to vision impairment. Traditional methods used for DR diagnosis have inherent limitations and are not conducive to large-scale rapid screening. Biomarkers can reflect the stage of the disease owing to their specificity and sensitivity， which is crucial for the early diagnosis of DR. In the present study， 142 potential literature-based biomarkers associated with DR were included in a knowledge-directed strategy. And metabolites with different physicochemical properties were chromatographically separated using a 100-mm Discovery HS F5 column. A pseudo-targeted metabolomics method based on ultra-high performance liquid chromatography-tandem mass spectrometry （UHPLC-MS/MS） that simultaneously scans positive and negative ions was established to improve analysis coverage and throughput. The method was validated using eight representative isotope-labeled internal standards as analytical targets. All isotope-labeled internal standards exhibited satisfactory linearities in both positive- and negative-ionization modes， with linear dynamic ranges spanning over three orders of magnitude and correlation coefficients （*r*²） above 0.995. Extraction recoveries ranged between 75% and 108% at three distinct concentration levels with relative standard deviations （RSDs） below 13%. Notably， 91% of the isotope-labeled internal standards exhibited intra-day precision with RSDs of less than 5% across both ionization modes. Similarly， 91% of the analytes demonstrated inter-day precision with RSDs of less than 10%， with all below 16.3%， indicating good method precision. The developed method was used to analyze 137 serum samples， including 40 DR-free patients with diabetes mellitus （NDR） and 97 patients with DR to investigate the practicality of the method. Quality control （QC） samples were used to evaluate the data， which revealed that the instrument was stable during the analytical sequence. Partial least squares discriminant analysis （PLS-DA） models were constructed to identify and differentiate between the metabolic profiles of the DR and NDR groups with the aim of providing a statistical basis for the classification and diagnosis of DR based on metabolic differences observed in the serum samples. The NDR group and DR groups of varying clinical grades were well separated. A total of 85 differential metabolites were identified between NDR and DR groups using nonparametric tests. Further analysis led to the selection of choline and 12-hydroxyeicosatetraenoic acid （12-HETE） as two markers that effectively distinguished the DR and NDR groups. In addition， the markers exhibited a good ability to distinguish between DR and NDR patients when used in combination. The pseudo-targeted metabolomics-based knowledge-directed method developed in this study provides a reference for screening and diagnosing DR.

糖尿病视网膜病变（diabetic retinopathy，DR）是由糖尿病引起的一种常见且严重的眼部并发症，具有高发病率和高致盲率，是全球成年人获得性视力丧失的主要原因之一^［1，2］^。我国约有1 950万DR患者，其中约1/5处于威胁视力的阶段，给个人和医疗系统带来沉重负担^［3］^。根据是否有新血管生成，将DR分为非增殖性DR和增殖性DR（PDR），非增殖性DR进一步分为轻度DR（NPDR）、中度DR（MNPDR）和重度DR（SNPDR）^［1］^。DR早期往往缺乏明显症状，导致患者常在视力受损后才就医，此时多已错过了最佳的治疗窗口，有时甚至造成失明。

早期诊断与及时干预对DR患者至关重要，可有效避免视力严重下降。临床上传统的DR检查方法包括眼底镜检查、眼底照相、荧光素眼底血管造影（fluorescein fundus angiography，FFA）和光学相干断层扫描等^［4-6］^。然而，这些方法存在局限性：眼底镜检查及眼底照相只能定性观察视网膜形态学变化，主观性强且无法发现早期病变；FFA 虽然是DR诊断的“金标准”，但检测时间长、具有侵入性，对患者全身情况有限制，且人力、物力消耗大，不利于大规模快速筛查。

近年来，基于人工智能（AI）的眼底诊断方法逐渐兴起。例如，Dai等^［7］^开发的深度学习系统DeepDR可实现DR的全程诊断；Li等^［8］^提出的DeepDR-LLM（integrated image-based deep learning and language models for primary diabetes care）系统可为糖尿病患者提供管理建议并辅助诊断DR。尽管AI技术为DR的快速诊断提供了新思路，但目前的算法主要依赖眼底图片，仍需专业人员监督，且在医疗资源匮乏地区难以推广^［9］^。因此，探索一种快速、高效、简便且低成本的体外诊断标志物对于DR的早期发现和诊断具有重要意义。

DR的发病机制尚不清楚，但近年来的研究显示，基因表达异常、表观遗传改变、信号通路失调及代谢紊乱均与DR的发生发展密切相关，尤其是代谢紊乱在DR进程中发挥关键作用。DR患者体内多条代谢通路（如脂质代谢、氨基酸代谢和能量代谢）发生显著变化。尽管现有的非靶向和靶向代谢组学研究已发现上百种差异代谢物，但目前仍缺乏明确的早期诊断生物标志物，难以满足大规模人群的DR筛查需求。

本研究建立了一种基于知识导向（结合已报道DR相关文献和标志物进行分析）的拟靶向代谢组学分析方法，用于DR的研究。首先，通过文献调研筛选出142个潜在的DR生物标志物；其次，根据这些候选标志物的物理化学性质，开发了一种基于超高效液相色谱-串联质谱（UHPLC-MS/MS）正、负离子切换扫描的分析方法，并对其进行了方法学验证；最后，将该方法应用于DR患者的血清样本分析，以筛选出能够有效区分DR的组合标志物。

## 1 实验部分

### 1.1 主要仪器、试剂与材料

Exion LC AD 高效液相色谱仪-QTRAP 6500+三重四极杆复合线性离子阱质谱仪（美国AB SCIEX公司）；Vanquish超高效液相色谱-Q Exactive高分辨质谱联用仪（美国Thermo公司）；冷冻离心浓缩仪（北京吉艾姆仪器有限公司）；台式高速冷冻离心机（日本HITACHI公司）；恒温混匀仪（德国Eppendorf公司）。

甲醇（色谱纯）购于Merck公司；甲酸（色谱纯）购于上海吉至生化科技有限公司；乙酸铵购自Sigma-Aldrich，超纯水由Milli-Q系统制备；代谢组学的14种内标和88种化学标准品购自Sigma-Aldrich公司、天津阿尔塔科技有限公司和上海甄准生物科技有限公司等。

### 1.2 样品采集

本研究涉及的137例血清样本来自上海市糖尿病综合防治体系研究（SIDPCSS），采集受试者的性别、年龄、糖尿病持续时间、血糖等信息。经上海市第六人民医院伦理委员会批准（批准号2018-KY-066（K）），所有参与者均签署了书面知情同意书。

### 1.3 样品前处理

将血清样本在冰上解冻并涡旋混匀后，取50 μL移入Eppendorf离心管中，加入200 μL含14种内标（质量浓度见表1）的甲醇提取剂。涡旋1 min后，在10 °C下以14 000 r/min离心10 min，取上清液进行冻干。冻干的样品加入50 μL含25%（体积分数）甲醇的水溶液复溶，涡旋并离心后取上清液用于 LC-MS 分析。从每个样本中取10 μL混合制备质量控制样本（quality control samples，QC），用于监测分析过程的稳定性，QC样本的预处理方法与实际样本相同。

**表 1:** 内标化合物的信息

IS	ESI modes	Class	Mass concentration/（μg/mL）
L-Phenyl-d5-alanine	+， -	amino acid	4
Glutamine-d5	+	amino acid	4
Cholic acid-2，2，4，4-d4	+， -	bile acid	2
Succinate-^13^C_4_	-	carboxylic acid	1
Folic acid-^13^C_5_	+， -	vitamin B	0.1
SM （d18：1/18：0）	+	SM	2
Stearic acid-18，18，18-d3	-	fatty acid	2
Palmitic acid-16，16，16-d3	-	fatty acid	2
Chenodeoxycholic acid-d4	+， -	bile acid	1.5
Hippuric acid-d5	+	benzenoid	0.4
Choline-d4	+	amine	2
12-HETE-d8	-	fatty acid	0.1
L-Leucine-5，5，5-d3	+	amino acid	1
2-Piperidone-d2	+	others	0.4

SM： sphingomyelin； HETE： hydroxyeicosatetraenoic acid.

### 1.4 分析条件

色谱柱：Discovery HS F5（100 mm×2.1 mm， 3 μm）；柱温：40 ℃；流速：0.3 mL/min；流动相A：含0.1%（体积分数）甲酸和2 mmol/L乙酸铵的水溶液；流动相B：含0.1%（体积分数）甲酸和2 mmol/L乙酸铵的甲醇溶液；进样量：2 μL。梯度洗脱程序：起始梯度为5%B，维持1 min，在3 min内增至60%B，随后在4 min内线性增加到90%B并维持4 min，在12.1 min处降回初始梯度5%B，平衡3.9 min。

离子源：电喷雾离子源（ESI），正负离子切换扫描；采集模式：多反应监测模式（MRM）；正离子的喷雾电压为5.5 kV；负离子的喷雾电压为-4.5 kV；气帘气（CUR）：276 kPa（40 psi）；离子源气体1：345 kPa（50 psi）；离子源气体2：345 kPa（50 psi）；离子源温度：400 ℃。

### 1.5 数据处理和统计分析

使用Xcalibur软件获取代谢物的母离子、子离子、保留时间等信息。使用SCIEX OS软件对所有代谢物的色谱峰面积进行积分。利用同位素内标对代谢物校正后，进行统计学分析。

采用SIMCA 13.0软件对校正后的数据进行主成分分析（PCA）和偏最小二乘法判别分析（PLS-DA），并通过交叉验证对PLS-DA模型进行评价。使用Multiple Experiment Viewer软件进行非参数检验，以寻找无糖尿病视网膜病变的糖尿病患者（NDR）与不同临床分级DR之间的差异代谢物。通过R语言构建热图和差异代谢物的受试者工作特征曲线（ROC曲线）。最后，使用GraphPad Prism 8.0软件绘制柱状图。

## 2 结果与讨论

### 2.1 知识导向的DR相关代谢物列表的确定

利用PubMed和Web of Science数据库，以“diabetic retinopathy”“metabolomics and diabetic retinopathy”“metabolomics and proliferative diabetic retinopathy”“metabolic profiling and diabetic retinopathy”等为关键词进行文献检索，检索时间范围截至2023年11月。将DR代谢相关的研究进行归纳总结，从样本类型为血液、玻璃体、泪液等人体生物样本的研究中，筛选出55篇相关文献，从中找出与DR和PDR相关的代谢物（表2）。将文献中满足以下任一条件的代谢物确定为候选代谢物：1）被重点讨论的潜在标志物；2）差异倍数（FC）>2或FC<0.5；3）变量权重值（VIP）>2的代谢物。进一步综合考虑DR相关重要代谢物被检测到的频次和生理学意义，从候选代谢物中筛选出142个代谢物，用于后续的拟靶向分析。

**表 2:** 糖尿病视网膜病变相关代谢物信息

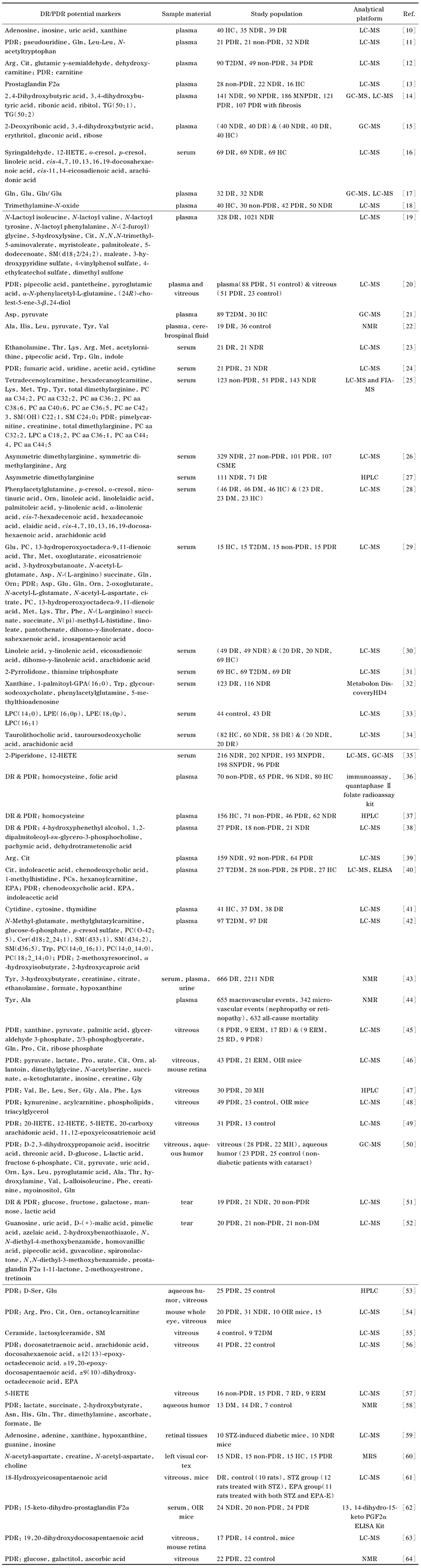

HC： healthy control； NDR： diabetes population without diabetic retinopathy； DR： diabetic retinopathy； LC-MS： liquid chromatography-mass spectrometry； PDR： proliferative diabetic retinopathy； Gln： glutamine； Leu： leucine； non-PDR： nonproliferative diabetic retinopathy； Arg： arginine； Cit： citrulline； T2DM： type 2 diabetes mellitus； TG： triglycerides； NPDR： mild nonproliferative diabetic retinopathy； MNPDR： moderate nonproliferative diabetic retinopathy； Glu： glutamic acid； Asp： aspartic acid； Ala： alanine； His： histidine； Tyr： tyrosine； Val： valine； NMR： nuclear magnetic resonance； Thr： threonine； Lys： lysine； Met： methionine； Trp： tryptophan； PC： phosphatidylcholine； LPC： lysophosphatidylcholine； FIA-MS： flow-injection analysis-mass spectrometry； CSME： clinically significant macular edema； HPLC： high performance liquid chromatography； Orn： ornithine； DM： diabetes mellitus； Phe： phenylalanine； LPE： lysophosphatidylethanolamine； SNPDR： severe nonproliferative diabetic retinopathy； EPA： eicosapentaenoic acid； ELISA： enzyme-linked immunosorbent assay； Cer： ceramide； Pro： proline； ERM： epiretinal membrane； RD： retinal detachment； OIR： oxygen-induced retinopathy； Val： valine； Ile： isoleucine； Ser： serine； Gly： glycine； MH： macular hole； MRS： magnetic resonance spectroscopy； Asn： asparagine； STZ： streptozotocin； EPA-E： eicosapentaenoic acid ethyl ester.

### 2.2 色谱-质谱条件优化

本研究纳入的142个DR相关代谢物涵盖了不同极性的多种代谢物，如氨基酸、胆汁酸、脂质等。为确保这些代谢物在选定的色谱条件下能够有效出峰且有较好的分离效果，对ACQUITY UPLC BEH C8（5 cm×2.1 mm， 1.7 μm）、Discovery HS F5（5 cm×2.1 mm， 3 μm）和Discovery HS F5（10 cm×2.1 mm， 3 μm）3种色谱柱进行了筛选和比较。结果表明，由于部分候选代谢物极性较大，在C8柱上集中在前3 min出峰，分离效果较差。相比之下，其在五氟苯基柱上有较好的分离，峰形也更为理想。进一步对比5 cm和10 cm长的Discovery HS F5色谱柱后，最终选择了10 cm的Discovery HS F5（10 cm×2.1 mm， 3 μm）色谱柱进行样本分析，以获得更好的分离效果。

为了增强离子化效率并调控pH，流动相中加入甲酸和乙酸铵。具体组成如下：A相为含0.1%（体积分数）甲酸和2 mmol/L乙酸铵的水溶液，B相为含0.1%（体积分数）甲酸和2 mmol/L乙酸铵的甲醇溶液。根据代谢物保留时间的分布，优化了液相色谱梯度和柱温。根据优化后的液相色谱条件，内标及142个目标代谢物的总离子流图见图1。

**图1:**
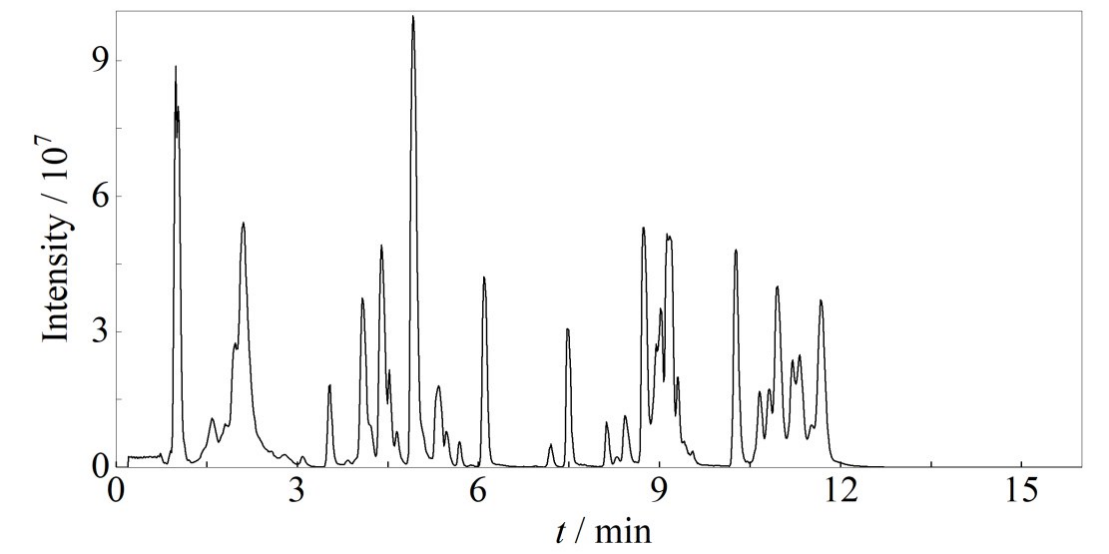
拟靶向方法中内标及目标代谢物在10 cm Discovery HS F5色谱柱的总离子流色谱图

根据目标代谢物的性质优化质谱条件。对于有标样的代谢物，采用标样优化代谢物的质谱参数，确定其保留时间、母离子、子离子、碰撞能（collision energy，CE）和去簇电压（declustering potential， DP）等。对于无标样的代谢物，首先利用高分辨质谱对QC样本进行分析，获取保留时间、MS^1^和MS^2^信息，结合数据库（如HMDB）的二级谱图，确定离子对并优化质谱参数。优化后的参数见表3。

**表 3:** 目标代谢物和内标化合物的质谱参数

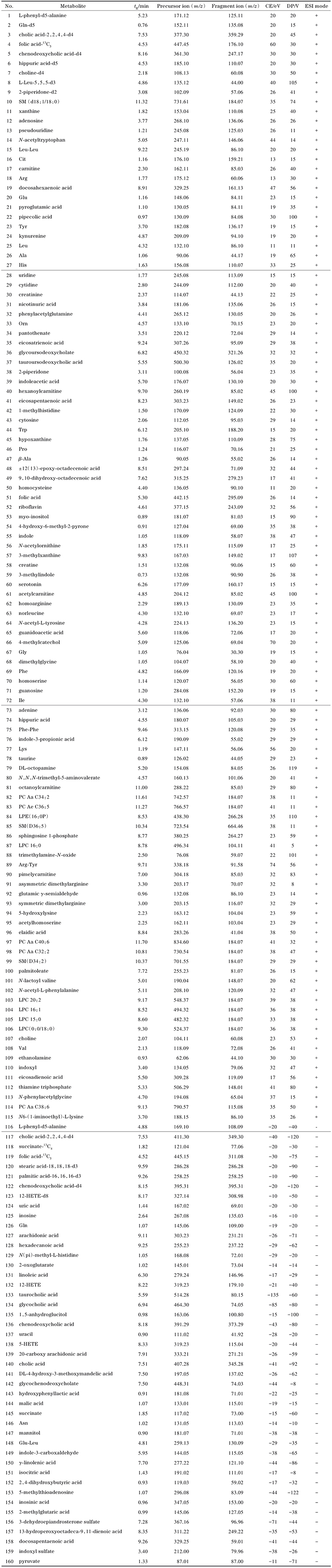

### 2.3 方法学评价

#### 2.3.1 线性关系和检出限

本研究所分析的代谢物主要包括氨基酸、氨基酸衍生物和胆汁酸等类别，选择涵盖氨基酸及其衍生物、胆汁酸、维生素、苯类有机物等类别的8种同位素内标（苯丙氨酸-d5（L-phenyl-d5-alanine）、胆酸-d4（cholic acid-d4）、鹅去氧胆酸-d4（chenodeoxycholic acid-d4）、马尿酸-d5（hippuric acid-d5）、亮氨酸-d3（L-leucine-d3）、2-哌啶酮-d2（2-piperidone-d2）、琥珀酸-^13^C_4_（succinate-^13^C_4_）和叶酸-^13^C_5_（folic acid-^13^C_5_））进行方法学考察，它们与待测物具有相似的化学性质和物理行为，且具有良好的稳定性。制备不同浓度的混合溶液，质量浓度由高到低依次为25 000、10 000、5 000、2 500、1 000、500、250、100、50、25、10、5、2.5、1、0.5、0.1 ng/mL。将不同浓度的混合标准溶液加入到血清中，每个水平平行处理3份，在优化方法下进行分析。以同位素内标的色谱峰面积作为纵坐标，对应的质量浓度为横坐标构建标准曲线，每个标准曲线至少包括6个浓度点。将同位素内标信噪比（*S/N*）≥3时的浓度设置为检出限（LOD）。如表4所示，8种内标的相关系数（*r* ^2^）都达到0.995及以上，均具有良好的线性关系，且线性范围在3个数量级以上。

**表 4:** 8个同位素内标在正、负离子模式下的线性关系和检出限

ESI mode	IS	LOD/（ng/mL）	Regression equation	Linear range/（ng/mL）	*r* ^2^
+	L-phenyl-d5-alanine	0.250	*y*=27137.734*x*-31396.400	5-10000	0.995
	cholic acid-d4	1.000	*y*=13291.700*x*-5673.280	2.5-10000	0.995
	chenodeoxycholic acid-d4	2.500	*y*=1865.577*x*-1962.004	5-5000	0.996
	hippuric acid-d5	0.250	*y*=16255.994*x*-2230.113	0.5-10000	0.995
	L-leucine-d3	5.000	*y*=269.239*x*-454.511	25-25000	0.998
	2-piperidone-d2	0.050	*y*=13600.611*x*+19335.051	5-10000	0.995
-	L-phenyl-d5-alanine	1.000	*y*=941.874*x*+124.198	2.5-25000	0.996
	cholic acid-d4	1.000	*y*=685.804*x*+48.025	5-25000	0.997
	succinate-^13^C_4_	2.500	*y*=1090.061*x*-565.412	25-25000	0.998
	folic acid-^13^C_5_	2.500	*y*=888.209*x*-1757.596	5-5000	0.995
	chenodeoxycholic acid-d4	0.050	*y*=5983.701*x*+987810	10-10000	0.998

*y*： peak area of the compound； *x*： mass concentration， ng/mL.

#### 2.3.2 提取回收率和精密度

制备低、中和高3个水平的同位素内标混合液，以考察提取回收率和精密度。其中，中等浓度参照内源性代谢物的浓度进行选取，低浓度和高浓度分别为中等浓度的一半和两倍。同位素内标的中等质量浓度分别为苯丙氨酸-d5 4 μg/mL、胆酸-d4 2 μg/mL、鹅去氧胆酸-d4 0.5 μg/mL、马尿酸-d5 0.4 μg/mL、亮氨酸-d3 4 μg/mL、2-哌啶酮-d2 1 μg/mL、琥珀酸-^13^C_4_ 1 μg/mL、叶酸-^13^C_5_ 0.5 μg/mL。分别将混合液在提取前和提取后加入到血清样品中，每个水平平行处理3份，单个样本进样两次（*n=*3×2），通过计算提取前后的内标色谱峰面积的比值进行回收率评估。在低、中、高3个浓度水平下，所有物质的提取回收率为75%~108%，相对标准偏差（RSD）均小于13%。

在一天内对同位素内标的低浓度、中等浓度和高浓度进行分析，每个水平平行处理3份，进样两次，以同一浓度下内标峰面积的RSD作为日内精密度。对于日间精密度的考察，连续3天预处理和进样，得到的内标峰面积的RSD作为日间精密度。在正、负离子模式下，同位素内标日内精密度的RSD小于5%的个数占总数的91%；日间精密度的RSD小于10%的个数占总数的91%，RSD最高不超过16.3%，说明该方法精密度良好（表5）。

**表 5:** 同位素内标化合物在低、中、高3个浓度水平下的精密度和提取回收率

ESI mode	IS	Intra-day RSDs/%			Inter-day RSDs/%			Extraction recoveries/% （Mean±RSD）		
		Low	Medium	High	Low	Medium	High	Low	Medium	High
+	L-phenyl-d5-alanine	1.4	3.5	1.8	4.4	5.1	3.1	99.7±8.0	89.3±4.0	93.7±1.9
	cholic acid-d4	4.4	4.5	0.7	7.9	7.1	7.9	99.6±8.1	84.1±11.7	89.1±3.3
	chenodeoxycholic acid-d4	2.8	1.6	2.3	5.6	5.5	6.5	96.6±7.0	84.6±5.5	87.3±5.6
	hippuric acid-d5	3.4	4.2	4.8	5.4	7.2	7.1	108.3±11.4	97.4±8.6	100.9±3.0
	L-leucine-d3	2.1	3.3	2.4	9.5	5.3	5.4	100.0±9.5	86.4±8.2	88.9±2.4
	2-piperidone-d2	4.7	3.2	3.0	15.1	6.9	7.4	87.7±10.1	82.3±3.3	81.2±5.5
-	L-phenyl-d5-alanine	2.8	1.7	1.6	6.7	6.1	7.5	103.0±8.2	89.3±4.5	91.9±4.0
	cholic acid-d4	3.6	1.0	4.2	7.2	7.1	8.0	99.3±8.5	85.5±5.5	89.6±7.7
	succinate-^13^C_4_	4.4	3.8	2.9	16.3	9.8	8.8	106.7±8.4	93.6±6.0	99.6±2.0
	folic acid-^13^C_5_	5.1	3.5	5.5	9.5	9.7	7.9	88.2±13.0	78.3±8.8	79.5±7.7
	chenodeoxycholic acid-d4	10.9	2.6	3.3	11.0	4.3	4.7	75.1±4.4	79.8±9.8	85±5

### 2.4 DR相关的差异代谢物筛查

#### 2.4.1 数据质量评价

以137例血清样本为示范性应用，利用所建立的拟靶向方法对样本进行分析，每10个样本进一针空白和QC。利用80%规则^［65］^和空白滤噪评估实际样本中142个代谢物的检出情况，最终有137个代谢物能够在本批样本中稳定检测。对稳定出峰的代谢物在QC中的重复性进行评价，如图2所示，有96.35%的代谢物采用内标校正后相对峰面积的RSD<30%，这些代谢物的峰面积总和占总峰面积的99.83%。对QC中RSD<30%的132个代谢物进行后续分析。主成分分析表明，QC样本紧密结合在一起，质量评价结果表明采集的数据稳定可靠，可用于后续分析。

**图2:**
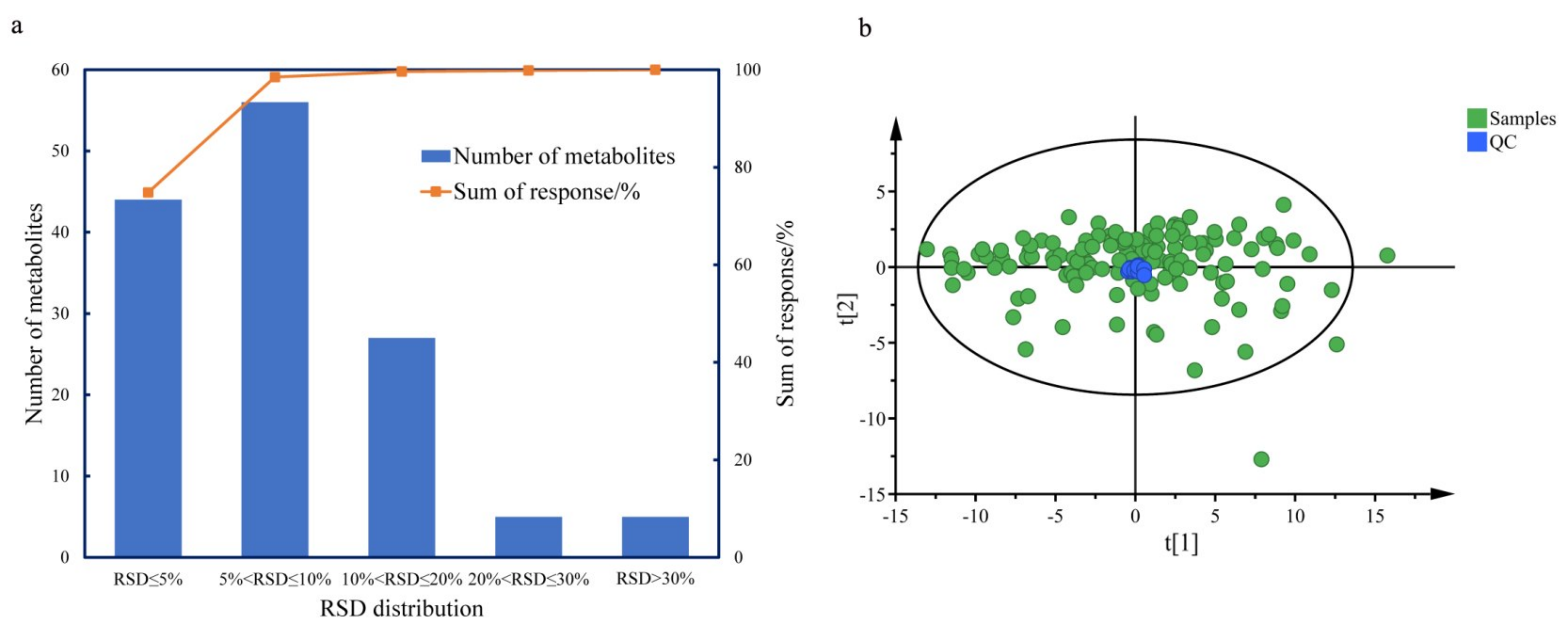
（a） 检测代谢物在QC中的相对标准偏差分布与（b）QC和实际样本的PCA图

#### 2.4.2 样本临床参数相关信息

本研究共分析了随机抽取的137名糖尿病患者，其中属于NDR的糖尿病患者有40位，DR有97位。根据临床表现，将DR患者分为NPDR（29）、MNPDR（33）、SNPDR（19）和PDR（16）患者。将患者临床参数总结在表6中，各组人群的年龄和BMI均无差异。

**表 6:** 研究对象的临床参数

Clinical parameter	Total	NDR	NPDR	MNPDR	SNPDR	PDR
Gender （male/female）	57/80	20/20	14/15	13/20	5/14	5/11
Age （years）	62.97±5.59	63.33±5.65	62.32±5.98	63.69±5.62	62.82±5.53	61.87±5.07
BMI （kg/m^2^）	25.49±3.54	25.58±3.43	25.04±3.51	25.19±3.01	25.60±4.01	26.61±4.47
SBP （mmHg）	140.89±20.11	129.18±11.93	143.83±21.36^**^	147.41±19.03^***^	145.35±22.99^*^	148.00±22.48^**^
DBP （mmHg）	81.00±9.71	80.92±7.30	81.97±11.87	81.13±10.92	82.06±8.31	77.64±10.05
FPG （mmol/L）	8.35±2.02	7.84±1.96	7.95±1.84	9.05±1.76^*^	9.71±2.41^*^	7.69±1.69
HbA1c （%）	7.62±1.39	6.91±1.18	7.50±1.47	8.28±1.25^***^	7.98±1.10^***^	8.09±1.45^**^
TC （mmol/L）	5.05±1.09	5.00±1.07	4.81±1.22	4.90±0.94	5.49±1.07	5.46±1.04
TG （mmol/L）	1.56±0.77	1.65±0.79	1.41±0.82	1.46±0.57	1.59±0.75	1.82±0.97
HDL-c （mmol/L）	1.35±0.32	1.29±0.28	1.35±0.31	1.45±0.37	1.38±0.32	1.33±0.35
LDL-c （mmol/L）	3.03±0.95	2.98±0.84	2.92±1.05	2.90±0.95	3.35±1.00	3.29±0.99
T2DM duration （years）	8.97±5.73	6.73±5.30	6.54±3.28	9.14±4.45^*^	13.53±6.57^***^	12.27±6.33^**^
Uric acid （μmol/L）	319.71±82.25	338.31±66.32	314.15±90.64	324.45±92.05	294.71±96.92	289.50±57.97^*^

BMI： body mass index； SBP： systolic blood pressure； DBP： diastolic blood pressure； FPG： fasting plasma glucose； HbA1c： hemoglobin a1c； TC： total cholesterol； HDL-c： high-density lipoprotein cholesterol； LDL-c： low-density lipoprotein cholesterol. * *p<*0.05， ** *p<*0.01， *** *p<* 0.001 compared to NDR. Data represent mean±SD.

#### 2.4.3 NDR与不同临床分级DR的代谢差异分析

为探究NDR与不同临床分级DR间的代谢变化，首先构建多变量PLS-DA模型。结果表明，NDR与不同临床分级DR之间有明显的分离趋势（图3）。对4个模型进行200次交叉验证，表明模型未拟合（图4）。

**图3:**
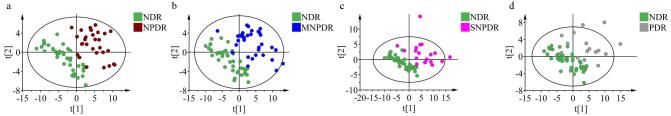
NDR分别与（a）NPDR、（b）MNPDR、（c）SNPDR、（d）PDR的PLS-DA得分图

**图4:**
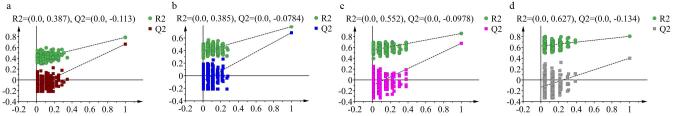
200次扰动测试得到的NDR分别与（a）NPDR、（b）MNPDR、（c）SNPDR和（d）PDR的交叉验证图

进一步采用Mann-Whitney U非参数检验筛选NDR与不同临床分级DR患者间的差异代谢物。NDR与NPDR间有78个差异代谢物，与MNPDR之间有77个差异代谢物，与SNPDR之间有77个差异代谢物，与PDR之间有38个差异代谢物。其中，NDR和不同临床分级DR间共有的差异代谢物有30个。以热图的形式展示共有的30个差异代谢物（*p*<0.05）的变化规律，从图5可以看到，与NDR相比，DR患者体内的精氨酸、组氨酸、鸟氨酸等氨基酸含量升高，此外，二十碳五烯酸、12-HETE和5-羟基二十碳四烯酸（5-HETE）等多不饱和脂肪酸及相关代谢产物明显增多。进一步结合错误发现率（FDR）寻找显著性差异代谢物，12-HETE、5-HETE、胆碱、二十碳五烯酸、*N*-乙酰-L-酪氨酸、溶血磷脂酰胆碱（LPC，0：0/18：0）、丙酮酸和鞘氨醇-1-磷酸等8个代谢物在NDR和4个临床分级中均有显著性差异（*p<*0.05且FDR<0.05）。

**图5:**
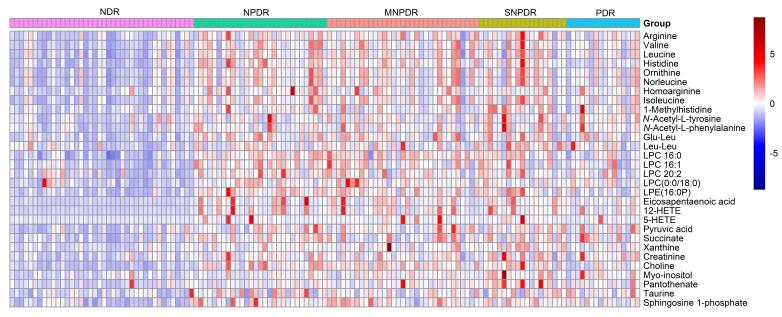
NDR与不同临床分级间共有的显著性差异代谢物热图

#### 2.4.4 NDR与DR的差异代谢物分析

进一步比较NDR和DR的代谢变化，两组间共有85个差异代谢物，涵盖脂质、氨基酸、核苷酸、酚类、吲哚类等多种代谢物类别，其中花生四烯酸、12-HETE、LPC和3-甲基黄嘌呤等代谢物在DR组显著增加。对其中32个显著差异代谢物（FC>1.5、*p*<0.05且FDR<0.05）结合二元逻辑回归逐步向前筛选组合标志物，同时校正性别、年龄及BMI等混杂因素，筛选到胆碱和12-HETE作为组合标志物。构建组合标志物胆碱和12-HETE的ROC曲线，如图6所示，相比于NDR，胆碱和12-HETE在DR中的含量上升。组合标志物的AUC值为0.973，敏感度和特异性分别是0.876、0.975，说明胆碱和12-HETE能很好地区分NDR和DR。此外，进一步将组合标志物用于NDR和非增殖性DR的分析。ROC结果显示敏感度和特异性分别为0.889和1，说明组合标志物能有效判别早期DR（AUC=0.990），对DR进行早期诊断具有重要意义。

**图6:**
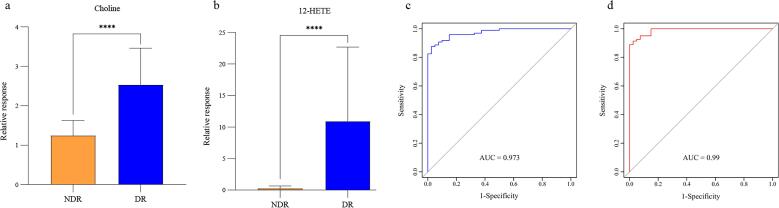
（a）Choline和（b）12-HETE在NDR和DR中的含量分布；组合标志物判别（c）NDR和DR、（d）NDR和早期DR的ROC曲线

对胆碱和12-HETE在不同临床分级DR中的浓度进行分析，发现两者在不同临床分级DR中均显著高于NDR。胆碱是一种重要的代谢物，根据KEGG的通路分析，胆碱涉及甘氨酸、丝氨酸和苏氨酸代谢、磷壁酸生物合成、甘油磷脂代谢和癌症中的胆碱代谢等多个代谢通路。胆碱的代谢产物三甲胺-*N*-氧化物（trimethylamine *N*-oxide，TMAO）等会影响炎症，可能促进糖尿病视网膜病变的发展。Xuan等^［35］^检测到胆碱在NDR和DR之间有显著性差异，DR患者体内的胆碱含量增多。有文献报道了喂食胆碱的小鼠产生代谢产物TMAO，TMAO会促进血管炎症^［66，67］^，而血管炎症是DR产生机制之一。Liu等^［68］^发现胆碱摄入量较多的女性患DR的几率更高。12-HETE是一种促炎的不饱和脂肪酸代谢物，可增加NADPH（nicotinamide adenine dinucleotide phosphate）氧化酶和活性氧（ROS）的合成，引起氧化应激，进而诱发视网膜内皮炎症^［69，70］^。12-HETE与花生四烯酸代谢、卵巢类固醇生成及醛固酮合成和分泌等通路相关。已有多篇文献报道12-HETE在DR发生发展中产生重要作用^［35，70，71］^。Moustafa等^［72］^发现12-HETE可激活Müller胶质细胞、影响谷氨酸生成并引发炎症和氧化反应。Chen等^［73］^发现儿童DR患病风险与血清12-HETE水平密切相关。胆碱参与甘油磷脂代谢，通过一系列酶促反应转化为磷脂酰胆碱，而磷脂酰胆碱是细胞膜中含量最丰富的磷脂之一，对维持细胞膜的结构和功能具有重要作用。细胞膜磷脂的分解可释放花生四烯酸^［74，75］^，后者是合成12-HETE的前体物质。12-HETE和胆碱的代谢通路之间存在潜在关联，两者的联合使用可从不同角度反映疾病的复杂代谢改变，提高对疾病的诊断准确性。胆碱和12-HETE在人体内具有多种重要的生理作用，仍需要深入了解两者的机制和相互关系，以更好地理解DR的发病机制，为疾病早期诊断提供思路。

## 3 结论

本文面向糖尿病视网膜病变研究，开发了一种基于知识导向型的拟靶向代谢组学分析策略，用于确定与疾病相关的代谢物并建立分析方法。方法学考察结果表明，该方法能稳定且可靠地检测DR相关代谢物。将该方法应用于NDR和DR血清样本分析中，筛选出能有效区分两者的组合标志物12-HETE和胆碱。此外，该组合标志物对NDR和早期DR也具有良好的区分能力，为DR筛查和早期诊断提供了参考。本文提出的分析策略具有广泛的适用性和良好的分析效率，可推广至其他代谢性疾病，为疾病的早期诊断和精准治疗提供参考。
